# Metabolic activation of 2‐amino‐1‐methyl‐6‐phenylimidazo [4,5‐*b*]pyridine and DNA adduct formation depends on p53: Studies in *T* *rp53*(+/+),*T* *rp53*(+/−) and *T* *rp53*(−/−) mice

**DOI:** 10.1002/ijc.29836

**Published:** 2015-09-22

**Authors:** Annette M. Krais, Ewoud N. Speksnijder, Joost P.M. Melis, Rajinder Singh, Anna Caldwell, Gonçalo Gamboa da Costa, Mirjam Luijten, David H. Phillips, Volker M. Arlt

**Affiliations:** ^1^ Analytical and Environmental Sciences Division, MRC‐PHE Centre for Environment and HealthKing's College LondonLondonSE1 9NHUnited Kingdom; ^2^ Center for Health Protection, National Institute for Public Health and the Environment (RIVM)BilthovenMA3721The Netherlands; ^3^ Department of Human GeneticsLeiden University Medical CenterLeiden2300The NetherlandsRC; ^4^ Department of Cancer Studies and Molecular MedicineUniversity of LeicesterLeicesterLE2 7LXUnited Kingdom; ^5^ Mass Spectrometry Facility, King's College LondonLondonSE1 9NHUnited Kingdom; ^6^ Division of Biochemical ToxicologyNational Center for Toxicological ResearchJeffersonAR72079; ^7^ Annette M. Krais current address is: Division of Occupational and Environmental MedicineLund University221 85LundSweden

**Keywords:** tumor suppressor p53, heterocyclic aromatic hydrocarbon, PhIP, carcinogen metabolism, DNA adduct formation, cytochrome P450, sulfotransferases, mouse model, mass spectrometry

## Abstract

The expression of the tumor suppressor p53 can influence the bioactivation of, and DNA damage induced by, the environmental carcinogen benzo[*a*]pyrene, indicating a role for p53 in its cytochrome P450 (CYP)‐mediated biotransformation. The carcinogen 2‐amino‐1‐methyl‐6‐phenylimidazo[4,5‐*b*]pyridine (PhIP), which is formed during the cooking of food, is also metabolically activated by CYP enzymes, particularly CYP1A2. We investigated the potential role of p53 in PhIP metabolism *in vivo* by treating *Trp53(+/+)*, *Trp53(+/−)* and *Trp53(−/−)* mice with a single oral dose of 50 mg/kg body weight PhIP. *N*‐(Deoxyguanosin‐8‐yl)‐2‐amino‐1‐methyl‐6‐phenylimidazo[4,5‐*b*]pyridine (PhIP‐C8‐dG) levels in DNA, measured by liquid chromatography‐tandem mass spectrometry, were significantly lower in liver, colon, forestomach and glandular stomach of *Trp53(−/−)* mice compared to *Trp53(+/+)* mice. Lower PhIP‐DNA adduct levels in the livers of *Trp53(−/−)* mice correlated with lower Cyp1a2 enzyme activity (measured by methoxyresorufin‐O‐demethylase activity) in these animals. Interestingly, PhIP‐DNA adduct levels were significantly higher in kidney and bladder of *Trp53(−/−)* mice compared to *Trp53(+/+)* mice, which was accompanied by higher sulfotransferase (Sult) 1a1 protein levels and increased Sult1a1 enzyme activity (measured by 2‐naphthylsulfate formation from 2‐naphthol) in kidneys of these animals. Our study demonstrates a role for p53 in the metabolism of PhIP *in vivo*, extending previous results on a novel role for p53 in xenobiotic metabolism. Our results also indicate that the impact of p53 on PhIP biotransformation is tissue‐dependent and that in addition to Cyp1a enzymes, Sult1a1 can contribute to PhIP‐DNA adduct formation.

## Abstract 2

**What's new?**

Many environmental carcinogens must be metabolized to form reactive electrophilic species capable of damaging DNA. The tumour suppressor p53 was shown to influence the cytochrome P450 (CYP)‐mediated biotransformation of the environmental carcinogen benzo[*a*]pyrene. The suspected carcinogen 2‐amino‐1‐methyl‐6‐phenylimidazo[4,5‐*b*]pyridine (PhIP), which is formed during the cooking of food, is also metabolically activated by CYP enzymes. This study shows that expression of p53 influences the biotransformation of PhIP. Using a gene knockout mouse model, the authors show that the impact of p53 on PhIP‐DNA adduct formation is tissue‐dependent and that, in addition to cytochrome P450 enzymes, sulfotransferases contribute to the bioactivation of PhIP.

The environment is becoming increasingly polluted with chemicals that play an important role in many human cancers. Genotoxic carcinogens from the environment can damage DNA by binding covalently to it, which can lead to mutations. The tumor suppressor *TP53*, which encodes p53, plays a crucial role during cellular response to DNA damage by regulating various cellular processes such as cell cycle arrest, apoptosis and DNA repair.^1^ Under genotoxic stress, cellular levels of p53 protein increase *via* post‐transcriptional mechanisms and the ability of p53 to bind specific DNA sequences is activated. p53 function disabled by mutations in the *TP53* coding sequence might lead to the development of tumors.^2^

Many environmental carcinogens require metabolism (*e.g*., catalysed by cytochrome P450 [CYP] enzymes) to form reactive electrophilic species capable of covalently binding to DNA (*i.e*., DNA adduct formation). We found recently in human cells that p53 expression is linked to CYP1A1‐mediated metabolic activation of the environmental carcinogen benzo[*a*]pyrene (BaP).^3^ BaP‐DNA adduct levels in *TP53(−/−)* cells were significantly lower than in *TP53(+/+)* cells, which correlated with BaP‐induced CYP1A1 expression in these cells. Bypass of the need for metabolic activation by treating cells with BaP‐7,8‐dihydrodiol‐9,10‐epoxide (BPDE), the activated metabolite of BaP, resulted in similar adduct levels in all cell lines, regardless of p53 status. In contrast, in *in‐vitro* experiments BaP‐DNA adduct formation was higher in livers and kidneys of *Trp53(−/−)* mice compared to *Trp53(+/+)* mice, while treating with BPDE resulted in similar adduct levels in both mouse lines.^4^ The higher BaP‐DNA adduct levels in the livers of BaP‐treated *Trp53(−/−)* mice correlated with higher Cyp1a1 enzyme activity in these animals.

Heterocyclic aromatic amines (HAAs) are carcinogenic compounds formed during cooking of meat, fish and poultry.^5^ Among these HAAs is 2‐amino‐1‐methyl‐6‐phenylimidazo[4,5‐*b*]pyridine (PhIP), which is mutagenic in several screening assays^6^ and which induces colon, prostate and mammary gland tumors in rats, which correspond to the principal sites in humans associated with Western diet‐related cancer, implying that it could be a human carcinogen.^5^, ^7^ The International Agency for Research on Cancer (IARC) has classified PhIP as possibly carcinogenic to humans (Group 2B).^5^ HAAs, including PhIP, form DNA adducts after metabolic activation and CYPs (particularly CYP1A2 but also CYP1A1) are the most important enzymes in the initial oxidation of PhIP, leading to the formation of *N*‐hydroxy‐PhIP (*N*‐OH‐PhIP) (see Supporting Information Fig. 1).^6^ Further metabolism by *N*‐acetyltransferases (NATs) or sulfotransferases (SULTs) converts *N*‐OH‐PhIP into esters capable of undergoing heterolytic cleavage to produce a nitrenium ion, which is the ultimate reactive species that reacts with DNA.^8^ The major covalent DNA adduct detected *in vivo* resulting from exposure of experimental animals and humans to PhIP is *N*‐(deoxyguanosin‐8‐yl)‐2‐amino‐1‐methyl‐6‐phenylimidazo[4,5‐*b*]pyridine (PhIP‐C8‐dG).^9^

In this study, we investigated the impact of *Trp53* status on the bioactivation of PhIP in *Trp53(+/+)*, *Trp53(* ***+/−*** *)* and *Trp53(−/−)* mice. These gene knock‐out mouse models have been shown to be particularly useful for studying *Trp53* in carcinogenesis because p53 function is highly cell‐type specific.^2^ PhIP‐C8‐dG was detected in DNA and quantified using liquid chromatography‐tandem mass spectrometry (LC‐MS/MS) selected reaction monitoring (SRM) mode. Tissue‐specific expression and activity of xenobiotic‐metabolising enzymes (XMEs) involved in PhIP metabolism were compared with DNA adduct formation in the same tissue.

## Material and Methods

### Carcinogen and adduct standard

Synthesis of PhIP was performed at the Biochemical Institute for Environmental Carcinogens, Prof. Dr. Gernot Grimmer‐Foundation (Grosshansdorf, Germany) according to a method described previously.^10^ The synthesis of PhIP‐C8‐dG and [^13^C_10_]PhIP‐C8‐dG was performed as reported.^11^

### Animal treatment

All animal experiments were conducted in accordance with the law at the Leiden University Medical Center, Leiden, The Netherlands, after approval by the institutional ethics committee. *Trp53(+/+)*, *Trp53(+/−)* and *Trp53(−/−)* male C57BL/6 mice were generated, housed, and genotyped as reported.^4^, ^12^ Groups of male *Trp53(+/+)*, *Trp53(+/−)* and *Trp53(−/−)* mice (3 months old; 25–30 g; *n* = 4/group) were treated with a single oral dose of 50 mg/kg body weight (bw) of PhIP dissolved in corn oil following a treatment protocol used previously to study PhIP metabolism.^10^ Control mice (*n* = 4) received solvent (corn oil) only. Animals were killed 24 hrs after treatment and their tissues (liver, lung, kidney, colon, small intestine, bladder, forestomach and glandular stomach) were collected, snap‐frozen in liquid nitrogen, and stored at −80°C until further analysis.

### DNA adduct analysis by LC‐ESI‐MS/MS

DNA from whole tissue was isolated by a standard phenol‐chloroform extraction method. An LC‐ESI‐MS/MS method to detect and quantify PhIP‐C8‐dG adducts in DNA was adapted as described previously (see Supporting Information File 1).^11^

### Preparation of microsomal and cytosolic samples

Hepatic and renal microsomal and cytosolic fractions (*n* = 4) were isolated as described^4^ in Supporting Information File 1.

### Measurement of Cyp1a enzyme activity in hepatic and renal microsomes

Hepatic and renal microsomal samples were characterised for Cyp1a1/2 activity by determining 7‐ethoxyresorufin O‐deethylation (EROD) activity and for Cyp1a2 by determining 7‐methoxyresorufin O‐demethylation (MROD) activity.^13^ Cyp1a enzyme activity was also measured with 3‐cyano‐7‐ethoxycoumarin (CEC) as substrate.^14^ Detailed descriptions of methods are given in Supporting Information File 1.

### Expression of Sult1a1 and Nat1/2 protein by western blotting

Microsomal and cytosolic proteins were separated using NuPage 4–12% Bis‐Tris sodium‐dodecyl sulfate (SDS)‐polyacrylamide gels (Life Technologies), and Western blotting was carried out as reported previously.^4^, ^15^ Sult1a1 and Nat1/2 forms were detected with antisera raised in rabbit against bacterial inclusion bodies of human SULT1A or NAT2 (Supporting Information File 1).^14^

### Measurement of Sult1a enzyme activity in renal cytosols

Renal cytosolic samples were characterised for Sult1a activity by monitoring the formation of *p*‐nitrophenol from a 5′‐phosphoadenosine 3′‐phosphosulfate (PAPS)‐regenerating system (Supporting Information File 1).^16^

## Results

### PhIP‐induced DNA adduct formation in *Trp53(*+/+*)*, *Trp53(+/−)* and *Trp53(−/−)* mice

**Figure 1:**
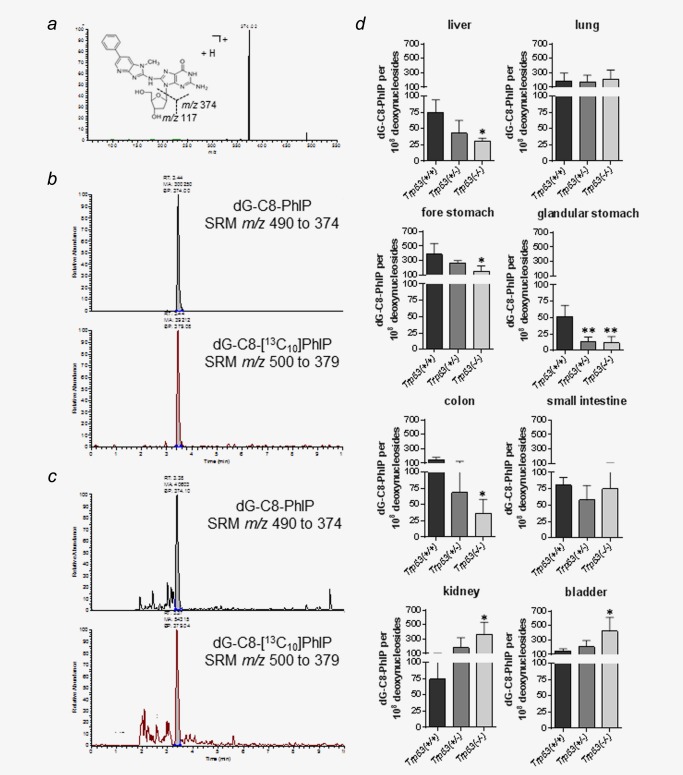
(*a*) Positive LC‐ESI‐MS/MS collision induced dissociation (CID) product ion spectrum of PhIP‐C8‐dG standard (1 ng/ml). The spectrum was acquired after continuous infusion at a flow rate of 10 μL/min with a syringe pump. Typical LC‐MS/MS SRM ion chromatograms for the determination of PhIP‐C8‐dG in (*b*) a mixture of PhIP‐C8‐dG standard (1 ng/ml) and 0.49 ng (1000 fmol) of [^13^C_10_]PhIP‐C8‐dG internal standard on column and in (*c*) 50 μg liver DNA isolated from *Trp53(+/+)* mice treated with 50 mg/kg bw PhIP and 0.49 ng (1000 fmol) of [^13^C_10_]PhIP‐C8‐dG internal standard on column. The SRM transitions monitored were *m/z* 490 to 374 for PhIP‐C8‐dG and *m/z* 500 to 379 for [^13^C_10_]PhIP‐C8‐dG. The analytical column was eluted isocratically at a flow rate of 200 μL/min with water/acetonitrile (0.1% formic acid) (85:15, *v*/*v*). (*d*) Quantitative LC‐ESI‐MS/MS analysis of PhIP‐C8‐dG in various tissues of *Trp53(+/+)*, *Trp53(+/−)* and *Trp53(−/−)* mice after exposure to PhIP. Values are the mean ± SD (*n* = 4). Statistical analysis was performed by one‐way ANOVA followed by Tukey post‐hoc test (*p < 0.05, **p < 0.01; different from *Trp53(+/+)* mice). [Color figure can be viewed in the online issue, which is available at wileyonlinelibrary.com.]

The formation of PhIP‐C8‐dG adducts was determined in various organs by LC‐MS/MS (Fig. 1). Figure 1a shows the LC‐ESI‐MS/MS collision induced dissociation (CID) product ion spectrum of PhIP‐C8‐dG standard whereas Figures 1b and 1c show typical LC‐MS/MS SRM ion chromatograms of the internal [^13^C_10_]PhIP‐C8‐dG alone or after spiking liver DNA isolated from *Trp53(+/+)* mice treated with 50 mg/kg bw PhIP, respectively. No PhIP‐C8‐dG was detectable in control (untreated) mice (data not shown). PhIP‐C8‐dG adduct levels were significantly lower in liver, forestomach, glandular stomach, and colon of *Trp53(−/−)* mice compared to *Trp53(+/+)* mice while adduct levels in kidney and bladder of *Trp53(−/−)* mice were significantly higher than in *Trp53(+/+)* mice (Fig. 1 *d*). For liver adduct levels were ∼2.1‐fold lower in *Trp53(−/−)* mice than in *Trp53(+/+)* mice whereas for kidney they were ∼4.8‐fold higher. Of note, no influence of p53 on PhIP‐DNA adduct formation was observed in *Trp53(+/−)* mice, except for glandular stomach in which similar adduct levels were found in *Trp53(+/−)* and *Trp53(−/−)* mice (Fig. 1 *d*).

### Expression of PhIP metabolising enzymes in *Trp53*(+/+), *Trp53*(*+/−*) and *Trp53*(*−/−*) mice

**Figure 2:**
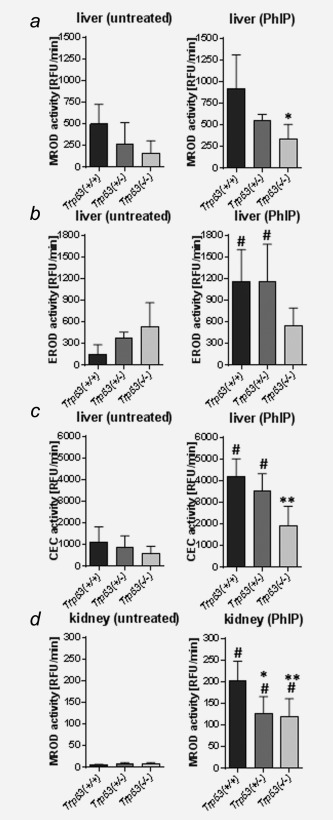
Measurement of microsomal enzymes metabolising PhIP in the livers and kidneys isolated from *Trp53(+/+)*, *Trp53(+/−)* and *Trp53(−/−)* mice. Cyp1a enzyme activity was measured as (*a*) MROD, (*b*) EROD as well as (*c*) CEC activity in hepatic microsomes and (*d*) MROD activity in renal microsomes isolated from control (untreated) mice (*left panel*) or mice treated with PhIP (*right panel*). Values are the mean ± SD of three independent determinations; 4 animals per genotype were analysed in three separate experiments. RFU, relative fluorescence unit. Statistical analysis was performed by one‐way ANOVA followed by Tukey post‐hoc test (^#^ *p* < 0.05, versus control [untreated] mice; *p < 0.05, **p < 0.01, different from PhIP‐treated *Trp53(+/+)* mice).

In rodents, CYP‐mediated *N*‐oxidation of PhIP occurs primarily in the liver which leads *via N*‐OH‐PhIP to the formation of PhIP‐C8‐dG in DNA. Metabolic activation of PhIP is catalysed mainly by CYP1A2, but also CYP1A1.^6^ Thus we studied Cyp1a1 and Cyp1a2 enzyme activity in hepatic microsomal fractions isolated from *Trp53(+/+), Trp53(+/−)* and *Trp53(−/−)* mice. MROD activity, which is considered a measure for Cyp1a2 enzyme activity, was significantly lower (∼2.7‐fold) in the liver of PhIP‐treated *Trp53(−/−)* mice relative to PhIP‐treated *Trp53(+/+)* mice (Fig. 2a) which correlates with the levels of PhIP‐DNA adducts in the livers of these animals. EROD and CEC activities are considered measures of Cyp1a activity, measuring both Cyp1a1 and Cyp1a2 enzyme activity. First, we found that PhIP treatment led to a significant induction of Cyp1a enzyme activity in livers of *Trp53(+/+)* and *Trp53(+/−)* mice, but not in *Trp53(−/−)* mice (Fig. 2b). Second, we observed that Cyp1a enzyme activity in PhIP‐treated *Trp53(−/−)* mice was lower than in *Trp53(+/−)* and *Trp53(+/+)* mice, although statistical significance was observed only in the samples measured with the CEC assay (Fig. 2c). Collectively these results show that lower hepatic Cyp1a enzyme activity in *Trp53(−/−)* mice correlates well with lower PhIP‐DNA adduct formation in the liver of these animals.

As *Trp53* status also impacted on PhIP‐DNA adduct formation in the kidney (compare Fig. 1 *d*) we next studied the expression and activity of PhIP metabolising enzymes in renal microsomal and cytosolic fractions. Due to the role of Cyp1a2 as major phase I enzyme activating PhIP we measured MROD activity in renal microsomes. We found that Cyp1a2 enzyme activity was strongly induced after PhIP treatment in all mouse lines (Fig. 2d). Interestingly, MROD activity was ∼two‐fold lower in *Trp53(+/−)* and *Trp53(−/−)* mice relative to *Trp53(+/+)* mice (Fig. 2d) which did not correlate with PhIP‐DNA adduct formation in this organ (compare Fig. 1 *d*).

**Figure 3:**
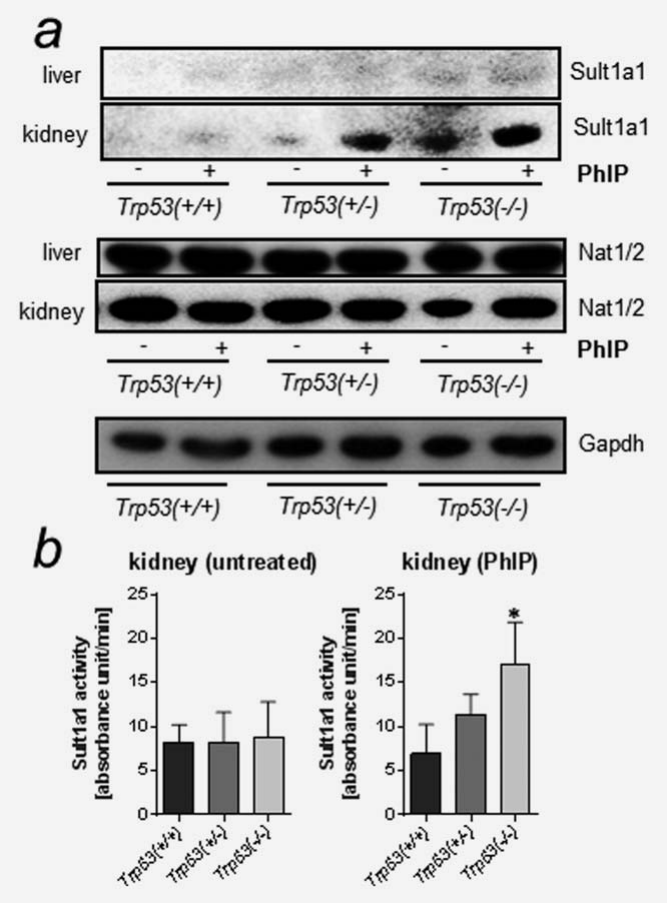
Measurement of cytosolic enzymes metabolising PhIP in the livers and kidneys isolated from *Trp53(+/+)*, *Trp53(+/−)* and *Trp53(−/−)* mice. (*a*) Western blot analysis of Sult1a1 and Nat1/2 in hepatic and renal cytosols. Representative images of the Western blotting are shown and at least duplicate analysis was performed from independent experiments. Gapdh protein expression was used as loading control for the cytosolic fractions and a representative blot is shown. (*b*) Sult1a enzyme activity was measured using a colorimetric assay with 2‐ naphthol. Values are the mean ± SD of three independent determinations; four animals per genotype were analysed in three separate experiments. Statistical analysis was performed by one‐way ANOVA followed by Tukey post‐hoc test (*p < 0.05, different from PhIP‐treated *Trp53(+/+)* mice).

Previous studies have shown that the Cyp1a‐mediated metabolite *N*‐OH‐PhIP can be further activated by phase II enzymes such as SULTs and NATs (see Supporting Information Fig. 1).^8^ SULTs and NATs can convert *N*‐OH‐PhIP into esters capable of undergoing heterolytic cleavage to produce a PhIP‐nitrenium ion which can bind covalently to DNA forming PhIP‐C8‐dG. Using Western blotting we found higher Sult1a1 protein levels in the kidney of *Trp53(−/−)* mice compared to *Trp53(+/+)* mice (Fig. 3 *a*). No such changes in Sult1a1 protein levels were observed in hepatic cytosolic samples, where there was no detectable expression. This finding was unexpected as liver has been reported to be a primary site of Sult1a1 expression.^17^ As the antibody was raised against bacterial inclusion bodies of human SULT1A, it is possible that the affinity to detect mouse Sult1a1 protein may be lower. However, differences in the organ‐specific distribution of Sults in mice have been described.^17^

No change in Nat1/2 protein levels were observed between mouse lines in hepatic and renal cytosolic samples (Fig. 3 *a*). This suggests that *Trp53* status does not modulate the expression of Nats in either liver or kidney of these mice. As *N*‐OH‐PhIP is activated by SULT1A enzymes (*i.e*., SULT1A1 and SULT1A2 in humans)^8^ we determined Sult1a1 enzyme activity in renal cytosols isolated from *Trp53(+/+), Trp53(+/−)* and *Trp53(−/−)* mice. We found that Sult1a enzyme activity was significantly increased in PhIP‐treated *Trp53(−/−)* mice compared to *Trp53(+/−)* and *Trp53(+/+)* mice exposed to PhIP (Fig. 3 *b*). This increase in Sult1a enzyme activity in *Trp53(−/−)* mice was in line with higher Sult1a1 protein levels (compare Fig. 3 *a*) and correlated with enhanced PhIP‐DNA adduct formation in the kidney of these animals (see Fig. 1 *d*).

## Discussion

In this study, we have used *Trp53(+/+)*, *Trp53(+/−)* and *Trp53(−/−)* mice to investigate the effect of p53 on PhIP‐DNA adduct formation *in vivo* and to examine the expression and activity of key enzymes involved in PhIP metabolism. We have conducted an *in vivo* study in order to take into account the roles of route of administration, absorption, renal clearance and tissue‐specific expression and activity of XMEs.^18^ PhIP‐DNA adduct formation was observed in all tissues investigated confirming a previous study involving multiple PhIP doses.^10^ However, the degree of DNA binding in various tissues of *Trp53(+/+)* mice was different between the two studies which is likely to be attributed to the different protocols (*i.e*., a single dose of 50 mg/kg bw PhIP versus five daily doses). Interestingly, PhIP‐induced DNA adduct formation was altered differently in multiple organs of *Trp53(−/−)* mice relative to *Trp53(+/+)* mice suggesting that the influence of p53 is highly tissue‐specific. Formation of PhIP‐C8‐dG adducts were significantly lower in liver, forestomach, glandular stomach and colon of *Trp53(−/−)* mice compared to *Trp53(+/+)* mice while adduct levels in kidney and bladder of *Trp53(−/−)* mice were significantly higher than in *Trp53(+/+)* mice. In lung and small intestine no difference in PhIP‐DNA adduct formation was observed between mouse lines.

Mainly, Cyp1a2, but also Cyp1a1, are considered to be the key enzymes responsible for *N*‐oxidation of PhIP to *N*‐OH‐PhIP,^6^ while *N*‐OH‐PhIP can be further activated by Nats and Sults.^8^ Our data showed that lower PhIP‐DNA adduct levels in the livers of *Trp53(−/−)* mice relative to *Trp53(+/+)* mice correlated with lower hepatic Cyp1a2 enzyme activity in the former animals. In contrast, higher PhIP‐C8‐dG levels in the kidney of *Trp53(−/−)* mice relative to *Trp53(+/+)* mice correlated with higher Sult1a1 protein levels and higher Sult1a enzyme activity in the former animals. Thus, our results not only confirm a previous study^4^ demonstrating an emerging role for p53 in Cyp‐mediated carcinogen metabolism *in vivo* but they also demonstrate the potential impact of p53 function on other XMEs (*i.e*., Sult1a1) in carcinogen metabolism *in vivo*.

Studies investigating carcinogen‐DNA adduct formation in transgenic mice with altered *Trp53* status are sparse.^4^, ^19^ Carmichael *et al*.^19^ observed a ∼twofold increase in diethylstilbestrol (DES)‐induced DNA adduct formation in *Trp53 (+/−)* mice compared to *Trp53(+/+)*, while *Trp53(−/−)* mice were not examined. The authors concluded that small differences in the protein expression of several Cyp enzymes could have contributed to the alterations in DES‐induced adduct levels.^19^ We recently found that DNA adduct formation induced by BaP was significantly higher in liver and kidney of *Trp53(−/−)* mice than *Trp53(+/+)* mice while 3‐nitrobenzanthrone (3‐NBA)‐induced DNA adduct formation was not altered in these mouse lines.^4^ These results could be explained by different metabolic pathways leading to DNA adduct formation. CYP1A1 is considered the key enzyme responsible for the metabolic activation of BaP^18^ while the most efficient enzyme to activate 3‐NBA is NAD(P)H:quinone oxidoreductase (NQO1).^20^ Higher BaP‐DNA adduct levels in the livers of *Trp53(−/−)* mice correlated with increased Cyp1a enzyme activity in these animals.^4^ In addition, significantly higher amounts of BaP metabolites were formed *ex vivo* in hepatic microsomes from BaP‐pretreated *Trp53(−/−)* mice compared to wild‐type animals.^4^ No differences were observed for 3‐NBA‐DNA adduct formation between *Trp53(+/+)*, *Trp53(+/−)* or *Trp53(−/−)* mice, which was in line with unchanged Nqo1 enzyme activity.^4^

In human cells, p53 has been shown to impact on CYP1A1‐mediated bioactivation of BaP, but not on the metabolic activation of 3‐NBA by NQO1.^3^, ^21^ In human cells, *in vitro* loss of p53 function (*i.e*., *TP53(−/−)* cells) resulted in considerably lower BaP‐DNA adduct levels compared to *TP53(+/+)* cells after BaP exposure.^3^, ^21^ We found that BaP‐induced CYP1A1 expression in human cells was regulated through p53 binding to a p53 response element (p53RE) in the regulatory region of CYP1A1,^3^ thereby enhancing its transcription. Others have found that in human cells p53 can induce the activity of CYP3A4 or UGT2B7 *via* its binding to p53REs and subsequent enhancement of gene transcription.^22^, ^23^ However, the role of p53 in CYP1A1‐mediated metabolism of BaP is different *in vitro* and *in vivo*.^3^, ^4^ While the induction of Cyp1a1 *via* p53 binding to a p53RE in the regulatory region of *Cyp1a1* fails to explain the impact of p53 on BaP metabolism in the liver *in vivo*,^4^ this mechanism could possibly be relevant for p53 function on Cyp1a2‐mediated PhIP metabolism in the liver (present study).

Our results indicate that Cyp1a2 does not have the same role in DNA adduct formation by PhIP in the kidney. Here, higher adduct levels in *Trp53(−/−)* mice than in *Trp53(+/+)* can be explained by enhanced expression and activity of Sults (*e.g*., Sult1a1). There is evidence of the expression of multiple forms of Sult enzymes in mouse kidney, mainly Sult1d1, but also Sult1a1, which have been linked to carcinogen‐DNA adduct formation in the kidney.^8^, ^24^ To our knowledge this is the first study that suggests that Sult‐mediated carcinogen activation can be linked to p53 function. The exact mechanism remains to be determined, but these results confirm an impact of p53 on both Phase I and Phase II enzymes.

In summary, we found that p53 functions influence the bioactivation of PhIP *in vivo*, indicating that gene‐environmental interactions need to be taken into account with regard to carcinogen metabolism. Results to date indicate that the cellular impact of p53 on carcinogen metabolism depends on the agent studied and the XMEs involved. Future investigations will need to assess whether other environmental carcinogens activated by Sults depend on p53 function and how p53 regulates the expression of XMEs *in vivo*.

## Supporting information

Supporting Information

Supporting Information
