# Supplementary material for: Metabolic activation of 2‐amino‐1‐methyl‐6‐phenylimidazo [4,5‐b]pyridine and DNA adduct formation depends on p53: Studies in T rp53(+/+),T rp53(+/−) and T rp53(−/−) mice
Source: Int J Cancer. 2015 Sep 22;138(4):976–82. doi: 10.1002/ijc.29836 (PMC4832306; doi:10.1002/ijc.29836)
Supplement: Supplementary file 1 — Supporting Information [file IJC-138-976-s001.pdf]

**Supplementary File 1**

## Material and Methods

### Carcinogen and adduct standard

Synthesis of PhIP was performed at the Biochemical Institute for Environmental Carcinogens, Prof. Dr. Gernot Grimmer-Foundation (Grosshansdorf, Germany) according to a method described previously.[1, 2] The synthesis of PhIP-C8-dG and [$^{13}C_{10}$]PhIP-C8-dG was performed as reported.[3]

### Animal treatment

All animal experiments were conducted in accordance with the law at the Leiden University Medical Center, Leiden, The Netherlands, after approval by the institutional ethics committee. *Trp53(+/+)*, *Trp53(+/-)* and *Trp53(-/-)* male C57BL/6 mice were bred, housed and genotyped as reported.[4, 5] Groups of male *Trp53(+/+)*, *Trp53(+/-)* and *Trp53(-/-)* mice (3 months old; 25-30 g; $n$ = 4/group) were treated with a single oral dose of 50 mg/kg body weight (bw) of PhIP following a treatment protocol used previously to study PhIP metabolism.[2] Control mice ($n$ = 4) received solvent (corn oil) only. Animals were killed 24 h after treatment and their liver, lung, kidney, colon, small intestine, bladder, forestomach, and glandular stomach was collected, snap-frozen in liquid nitrogen and stored at −80°C until further analysis.

### DNA adduct analysis by LC-ESI-MS/MS

DNA from whole tissue was isolated by a standard phenol-chloroform extraction method. To detect and quantify PhIP-C8-dG adducts in DNA a LC-ESI-MS/MS method was adapted as described previously.[3] Briefly, 0.49 ng (1000 fmol) of [$^{13}C_{10}$]PhIP-C8-dG internal standard (100 fmol/µL) was added to 50 µg of DNA samples, evaporated and redissolved in 150 µL of Tris Buffer (50 mM Tris, 0.1 mM EDTA, pH 7.1) and 1.5 µL of 1 M $MgCl_2$. Samples were first incubated with 15 µL of deoxyribonuclease I (2 mg/mL dissolved in 0.15 M NaCl, 10 mM $MgCl_2$) at 37°C for 6 h and further with 9.0 µL of snake venom phosphodiesterase I from *C. adamanteus* (0.001 U/µL dissolved in 0.11 M Tris-HCl, 0.11 M NaCl, 15 mM $MgCl_2$, pH 8.9) and 4.8 µL of alkaline phosphatase from *E. coli* (0.315 U/µL) at 37°C for 15 h. Samples were precipitated twice with methanol, centrifuged and the methanol extracts

were evaporated. The DNA was dissolved in 100 μL of HPLC grade water/methanol (0.1% formic acid) (50:50, *v*/*v*) and left for 2 h at –20°C to precipitate impurities.

For LC-ESI-MS/MS analysis a Thermo Accela LC system interfaced directly to Thermo TSQ Access triple quadrupole mass spectrometer (Thermo, Hemel Hempstead, UK) was used. Sample separation was performed on a Hypersil GOLD™ C18 reverse phase column, (50 × 2.1 mm, 1.9 μm; Thermo Scientific) at 25°C. Operation conditions were: electrospray ionization (ESI) in positive ion mode; capillary temperature 350°C; source voltage 3.5 kV; collision gas argon; and collision energy 20 eV. A 10 μL aliquot equivalent to 50 μg of hydrolysed DNA containing 1000 fmol of dG-C8-[$^{13}C_{10}$]PhIP internal standard was injected onto the column. The flow of the first 2 minutes was diverted directly into the waste. The isocratic flow was maintained at flow rate of 200 μL/min with water/acetonitrile (0.1% formic acid) (85:15, *v*/*v*) for 8.0 min. The total run time was 10 min.

The hydrolysed DNA samples were analyzed in positive ESI MS/MS SRM mode for $[M + H]^+$ ion to adducted base $[B + 2H]^+$ transition of *m/z* 490 to 374 for PhIP-C8-dG and *m/z* 500 to 379 for [$^{13}C_{10}$]PhIP-C8-dG. The level of the adduct in the DNA sample was determined from the ratio of the peak area of the [$^{13}C_{10}$]PhIP-C8-dG internal standard and is expressed as adducts per $10^8$ deoxynucleosides. The calibration curves were constructed by the addition of different amounts of the unlabelled PhIP-C8-dG standard (ranging from 0.2 to 1000 fmol) plus 1000 fmol of the [$^{13}C_{10}$]PhIP-C8-dG internal standard to 50 μg of hydrolysed calf thymus DNA as the matrix.

**Preparation of microsomal and cytosolic samples**

Hepatic and renal microsomal and cytosolic fractions ($n$ = 4) were isolated as described.[5] Briefly, the frozen tissue was homogenised in a Qiagen TissueLyser II (Hilden, Germany) in 0.067 M potassium phosphate buffer (pH 7.4) with 0.5% KCl. Nuclei and debris were removed by centrifugation at 18,000 g for 30 minutes at 4ºC. The supernatant was centrifuged at 100,000 g for 1 h yielding a pellet (i.e. microsomal faction) and supernatant (i.e. cytosolic fraction). Protein concentrations in cytosolic and microsomal fractions were measured using the bicinchoninic acid (BCA) protein assay with bovine serum albumin as a standard.

**Measurement of Cyp1a enzyme activity in hepatic and renal microsomes**

Hepatic and renal microsomal samples were characterised for Cyp1a1/2 activity using 7-ethoxyresorufin O-deethylation (EROD) activity and for Cyp1a2 using 7-methoxyresorufin

O-demethylation (MROD) activity.[6] The fluorescent signal (measured as relative fluorescence unit [RFU]/minute) was measured on a Synergy HT Plate Reader (Bio-TEK Instruments, USA; 530 nm excitation, 580 nm emission). Cyp1a enzyme activity (as relative RFU/minute) was also measured with 3-cyano-7-ethoxycoumarin (CEC) as substrate.[7] Briefly, in a 96-well plate the incubation mixture (200 µL) contained 67 mM sodium phosphate buffer (pH 7.4), 9 mM glucose-6-phosphate, 0.9 U glucose-6-phosphate dehydrogenase, 4.5 mM $MgCl_2$, 0.9 mM NADP, 5 µM CEC (dissolved in DMSO) and 50 µg of microsomal fraction. The reaction was initiated by the addition of CEC and the formation of 3-cyano-7-hydroxycoumarin was measured every 2 min for 30 min (409 nm excitation, 460 nm emission).

**Expression of Sult1a1 and Nat1/2 protein by Western blotting**

Microsomal and cytosolic proteins (10-20 µg) were separated using NuPage 4-12% Bis-Tris sodium-dodecyl sulfate (SDS)-polyacrylamide gels (Life Technologies), and Western blotted as previously reported.[5, 8, 9] Sult1a1 and Nat1/2 forms were detected with antisera raised in rabbit against bacterial inclusion bodies of human SULT1A or NAT2.[10, 11] These antibodies were a generous gift from Hansrüdi Glatt (German Institute for Human Nutrition, Nuthetal, Germany). In cytosolic samples anti-SULT1A and anti-NAT antisera were used at 1:10,000 and peroxidase-conjugated goat anti-rabbit antibody (CST7074, Cell Signalling Technology, 1:10,000) was used as secondary antibody. Previous studies have shown that anti-SULT1A and anti-NAT antisera have some cross-reactivity, detecting human SULT1A1 and SULT1A3 or human NAT1 and NAT2, respectively.[7]

**Measurement of Sult1a enzyme activity in renal cytosols**

Renal cytosolic samples were characterized for Sult1a activity by monitoring the formation of *p*-nitrophenol from a 5'-phosphoadenosine 3'-phosphosulfate (PAPS)-regenerating system.[12] In this assay, sulfotransferase catalyzes the synthesis of 2-naphthylsulfate from 2-naphthol and PAPS, while continuously regenerating PAPS by using *p*-nitrophenyl sulfate as a sulfo group donor. Briefly, in a 24-well plate the incubation mixture (total 1 ml) contained 67 mM sodium phosphate buffer (pH 7.4), 20 µM PAPS, 5 mM p-nitrophenyl sulfate potassium salt, 5 mM $MgCl_2$ and 100 µg of cytosolic fraction. The reaction was initiated by adding 2-naphtol (final concentration 0.1 mM). The colorimetric formation of *p*-nitrophenol was measured (as relative absorbance unit/minute) at 405 nm every 2 min for 10 min on a Synergy HT Plate Reader (Bio-TEK Instruments).

**References Supplementary File**


1. Lindstrom S. Friedlander synthesis of the food carcinogen 2-amino-1-methyl-6-phenylimidazo[4,5-b]pyridine. *Acta Chem Scand* 1995;**49**: 361-3.
2. Arlt VM, Singh R, Stiborova M, Gamboa da Costa G, Frei E, Evans JD, Farmer PB, Wolf CR, Henderson CJ, Phillips DH. Effect of hepatic cytochrome P450 (P450) oxidoreductase deficiency on 2-amino-1-methyl-6-phenylimidazo[4,5-b]pyridine-DNA adduct formation in P450 reductase conditional null mice. *Drug Meta Dispos* 2011;**39**: 2169-73.
3. Singh R, Arlt VM, Henderson CJ, Phillips DH, Farmer PB, Gamboa da Costa G. Detection and quantitation of N-(deoxyguanosin-8-yl)-2-amino-1-methyl-6-phenylimidazo[4,5-b]pyridine adducts in DNA using online column-switching liquid chromatography tandem mass spectrometry. *J Chromatogr B Analyt Technol Biomed Life Sci* 2010;**878**: 2155-62.
4. Jacks T, Remington L, Williams BO, Schmitt EM, Halachmi S, Bronson RT, Weinberg RA. Tumor spectrum analysis in p53-mutant mice. *Curr Biol* 1994;**4**: 1-7.
5. Krais AM, Speksnijder EN, Melis JPM, Indra R, Moserova M, Godschalk RW, van Schooten FJ, Seidel A, Kopka K, Schmeiser HH, Stiborova M, Phillips DH, et al. The impact of p53 on DNA damage and metabolic activation of the environmental carcinogen benzo[a]pyrene: effects in Trp53(+/+), Trp53(+/-) and Trp53(-/-) mice. *Arch Toxicol* 2015; in press.
6. Mizerovska J, Dracinska H, Frei E, Schmeiser HH, Arlt VM, Stiborova M. Induction of biotransformation enzymes by the carcinogenic air-pollutant 3-nitrobenzanthrone in liver, kidney and lung, after intra-tracheal instillation in rats. *Mutat Res* 2011;**720**: 34-41.
7. Martin FL, Patel, II, Sozeri O, Singh PB, Ragavan N, Nicholson CM, Frei E, Meinl W, Glatt H, Phillips DH, Arlt VM. Constitutive expression of bioactivating enzymes in normal human prostate suggests a capability to activate pro-carcinogens to DNA-damaging metabolites. *Prostate* 2010;**70**: 1586-99.
8. Hockley SL, Arlt VM, Brewer D, Giddings I, Phillips DH. Time- and concentration-dependent changes in gene expression induced by benzo(a)pyrene in two human cell lines, MCF-7 and HepG2. *BMC Genomics* 2006;**7**: 260.
9. Kucab JE, Phillips DH, Arlt VM. Metabolic activation of diesel exhaust carcinogens in primary and immortalized human TP53 knock-in (Hupki) mouse embryo fibroblasts. *Environ Mol Mutagen* 2012;**53**: 207-17.
10. Muckel E, Frandsen H, Glatt HR. Heterologous expression of human N-acetyltransferases 1 and 2 and sulfotransferase 1A1 in Salmonella typhimurium for mutagenicity testing of heterocyclic amines. *Food Chem Toxicol* 2002;**40**: 1063-8.
11. Teubner W, Meinl W, Florian S, Kretzschmar M, Glatt H. Identification and localization of soluble sulfotransferases in the human gastrointestinal tract. *Biochem J* 2007;**404**: 207-15.
12. Frame LT, Ozawa S, Nowell SA, Chou HC, DeLongchamp RR, Doerge DR, Lang NP, Kadlubar FF. A simple colorimetric assay for phenotyping the major human thermostable phenol sulfotransferase (SULT1A1) using platelet cytosols. *Drug Meta Dispos* 2000;**28**: 1063-8.
