# Supplementary material for: Metabolic activation of 2‐amino‐1‐methyl‐6‐phenylimidazo [4,5‐b]pyridine and DNA adduct formation depends on p53: Studies in T rp53(+/+),T rp53(+/−) and T rp53(−/−) mice
Source: Int J Cancer. 2015 Sep 22;138(4):976–82. doi: 10.1002/ijc.29836 (PMC4832306; doi:10.1002/ijc.29836)
Supplement: Supplementary file 2 — Supporting Information [file IJC-138-976-s002.pdf]

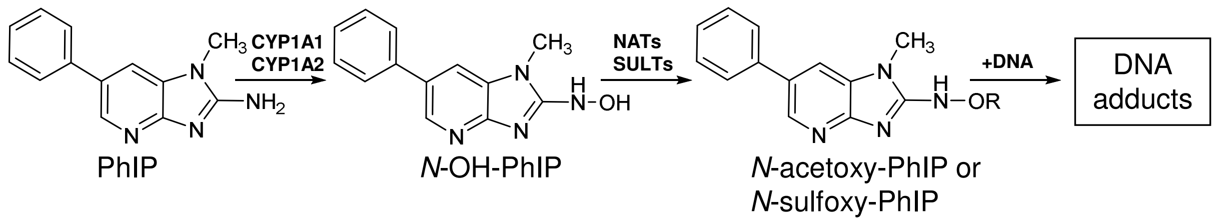

Supporting Figure 1. Metabolic activation of PhIP and DNA adduct formation. See text for details. R = –C(O)CH_3 or –SO_3H.
